# *AXIOME*: automated exploration of microbial diversity

**DOI:** 10.1186/2047-217X-2-3

**Published:** 2013-03-13

**Authors:** Michael DJ Lynch, Andre P Masella, Michael W Hall, Andrea K Bartram, Josh D Neufeld

**Affiliations:** 1 Department of Biology, University of Waterloo, 200 University Avenue West, Waterloo, Ontario, N2L 3G1, Canada

**Keywords:** Microbial ecology, Automation, SSU rRNA, High-throughput sequencing, QIIME, mothur

## Abstract

**Background:**

Although high-throughput sequencing of small subunit rRNA genes has revolutionized our understanding of microbial ecosystems, these technologies generate data at depths that benefit from automated analysis. Here we present AXIOME (Automation, eXtension, and Integration Of Microbial Ecology), a highly flexible and extensible management tool for popular microbial ecology analysis packages that promotes reproducibility and customization in microbial research.

**Findings:**

AXIOME streamlines and manages analysis of small subunit (SSU) rRNA marker data in QIIME and mothur. AXIOME also implements features including the PAired-eND Assembler for Illumina sequences (PANDAseq), non-negative matrix factorization (NMF), multi-response permutation procedures (MRPP), exploring and recovering phylogenetic novelty (SSUnique) and indicator species analysis. AXIOME has a companion graphical user interface (GUI) and is designed to be easily extended to facilitate customized research workflows.

**Conclusions:**

AXIOME is an actively developed, open source project written in Vala and available from GitHub (http://neufeld.github.com/axiome) and as a Debian package. Axiometic, a GUI companion tool is also freely available (http://neufeld.github.com/axiometic). Given that data analysis has become an important bottleneck for microbial ecology studies, the development of user-friendly computational tools remains a high priority. AXIOME represents an important step in this direction by automating multi-step bioinformatic analyses and enabling the customization of procedures to suit the diverse research needs of the microbial ecology community.

## Findings

### Rationale

Next-generation sequencing technologies have improved our ability to study complex microbial communities, but have also posed significant computational challenges associated with analyzing such large sequence datasets. The research community has developed multiple analysis platforms [1-4] to manage analysis of taxonomic high-throughput sequencing data, particularly for the study of microbial small subunit (SSU) rRNA sequence data. As implemented, these pipelines require extensive user intervention and are not particularly well suited to extension. To address these issues, we developed the Automation, eXtension, and Integration Of Microbial Ecology (AXIOME) package and the associated graphical user interface, Axiometic. AXIOME simplifies analyses common to native installs of multiple analysis platforms by using XML scripting and configuration. Generating AXIOME’s XML input using Axiometic, the companion GUI, simplifies scripting and further increases usability. AXIOME also extends functionality by offering several additional analytical plug-ins and easily enables the implementation of user-specific functionality for customized workflows.

### Functionality

#### Automation and checkpointing and reproducible research

Analysis environments, such as QIIME and mothur, have provided open source and effective tools for the analysis of high-throughput marker sequencing data (e.g., SSU rRNA). These environments are not easily automated beyond shell scripting, which is available for essentially any software installed on a Unix/Linux-based operating system. While effective, shell scripting can present a significant barrier to researchers using the software. AXIOME avoids this difficulty by interpreting commands from an XML-based input file containing simplified instructions and analysis blocks. This XML-based configuration file can be created manually, from workflow templates, or using the simple and interactive GUI tool, Axiometic.

Analysis pipelines for high-throughput marker data can consume many CPU hours due to dataset size and analysis complexity. Workflows can be interrupted for a myriad of reasons, including power failure and software error. Furthermore, analytical parameters can be modified or analyses added based on preliminary results. Built into AXIOME, using the make software package, is the ability to automatically restart a workflow from the last valid position, which does not unnecessarily reproduce previous valid results. This behavior can significantly reduce computational load and errors caused by excessive user intervention.

Efforts such as metadata standardization [5] and bioinformatic tools stressing reproducibility [6,7] represent attempts to make bioinformatic workflows documented and reproducible, thus facilitating collaboration. The use of XML-based instructions defining an entire AXIOME workflow has the added benefit of contributing to these reproducible research initiatives. This is a useful addition to both individual researchers and to the collaboration of different investigators, and can be packaged with research publications.

#### Extension

AXIOME manages QIIME [2] analyses and supports sequence processing and α-diversity in mothur [1]. In addition to offering common α- and β-diversity measures, there are several functions specific to AXIOME v.1.6. AXIOME enables the assembly and de-multiplexing of Illumina paired-end reads through the use of PAired-eND Assembler for Illumina sequences (PANDAseq [8]). Post-assembly analysis techniques unique to AXIOME include (i) non-negative matrix factorization [9], a technique that identifies overlapping patterns between samples, generating a concordance model, that can then be used for a non-negative factorization of the sample matrix (used to visualize the importance of specific taxa within a sample or a cluster of samples), (ii) multi-response permutation procedures (MRPP [10]), which tests for significant differences between sampling groups and the degree of within-group sample clustering, (iii) recovering and exploring phylogenetic novelty (SSUnique [11]) and (iv) indicator species analysis [12], a method for identifying operational taxonomic units (OTUs) that are significantly associated with user-defined sample treatment groups. Future releases of AXIOME will continue to incorporate analysis techniques reflecting advances in the analysis of marker data in ecology.

One advantage of the AXIOME package is the ability to quickly extend functionality to include new protocols, easily customizing research workflows. This also provides the opportunity to test alternative approaches to sequence analysis before implementing them in standard distributions of software environments, such as QIIME or mothur. In AXIOME, individual analyses are managed through the corresponding XML tag within the configuration file, and custom XML tags that invoke novel analyses can be built into the source to extend AXIOME. Full instructions and templates for extension are provided with the source and documentation. By facilitating extension of analysis pipelines, the implementation of previously existing ecological methods and the development of novel techniques can progress at a faster rate than through standard release schedules. This will foster experimentation and increase community involvement in these efforts.

#### AXIOME workflow

**Figure 1:**
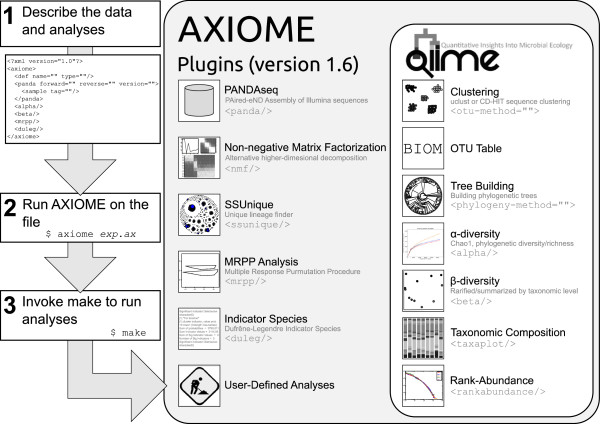
Schematic representation of the AXIOME workflow and its relation to existing QIIME analyses.

**Figure 2:**
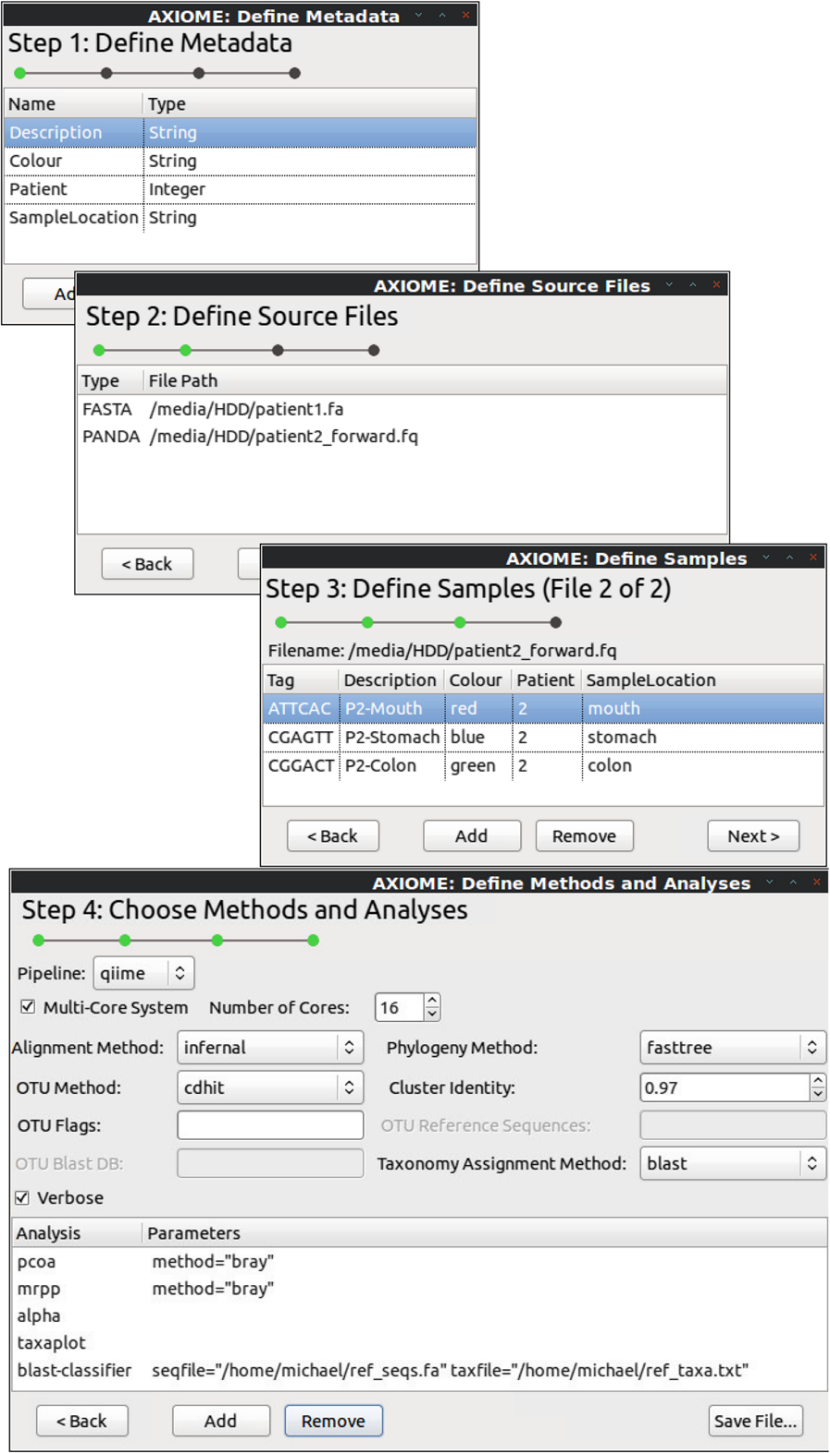
**Axiometic, a multi-platform companion GUI for AXIOME with four steps defining: metadata associated with samples, the data source files, expressions for sorting samples within source file and the analyses for the AXIOME pipeline to run.** The resulting XML control file is interpreted by AXIOME to run analyses. Axiometic is available for Linux, Windows and is in development for Mac OS X.

All user-defined workflow analyses and parameters are outlined in a single XML configuration file, which is processed by AXIOME, generating a Makefile that controls and manages analyses. By leveraging these tools, an entire run of QIIME and all requested extensions requires only three steps (Figure 1). Additionally, any interruption in processing can be circumvented by re-running the make command, which will act as a checkpoint by restarting from the last valid position. Furthermore, a companion GUI, Axiometic (Figure 2), allows users to easily construct this XML configuration file. Even though AXIOME is designed to work within a Linux environment, the XML configuration file can be generated on any system and transferred to the analysis environment. Axiometic is based on a platform-independent toolkit, further facilitating XML script generation.

Distributed with the source is a sample analysis, including instructions, XML workflow script, data files and expected output. The workflow management of AXIOME fosters reproducible, predictable and automated analyses, which are challenging goals given the large sequence datasets now being generated. AXIOME adds to the expanding computational toolbox supporting the next generation of research efforts in microbial ecology.

#### Comparison to related work

The Galaxy project [7,13,14] is a web-based analysis management and distribution system for biomedical research. Currently, analysis of SSU rRNA marker data within Galaxy can be accomplished through a mothur v.1.27.0 [1] implementation. Additionally, a QIIME [2] wrapper (QIIME-Galaxy) is under development for Galaxy. Within Galaxy, these implementations are intended to provide a managed workflow, either locally or within the cloud, which will be a suitable solution for many researchers. Besides analysis techniques specific to AXIOME, our software offers some features not present in either Galaxy-associated packages. For example, AXIOME uses a package manager, greatly simplifying installation (apt-get install). Furthermore, AXIOME excels at local management and extension of marker gene workflows with modular XML scripting and checkpointing, allowing for rapid exploration of parameter and analysis modifications. When marker gene workflows are more fully integrated into Galaxy, we envision AXIOME as a complementary system for workflow management and extension. To actively contribute to the open source analysis community, we are in the process of contributing to Galaxy the various analysis routines specific to AXIOME (Figure 1).

#### Availability of AXIOME

AXIOME is an actively developed, open source project written in Vala and available from GitHub (http://neufeld.github.com/axiome) and also as a Debian package, serving as a companion to any native QIIME (v.1.4 and above) or mothur install. Specific details of the design and implementation of AXIOME are packaged with the source, including detailed explanations of tool development and the relationship between files in the repository. Axiometic, a GUI companion tool for easily generating AXIOME XML analysis instructions, is also freely available (http://neufeld.github.com/axiometic). AXIOME is compatible with the .biom universal data file format [15] and was designed to work within a Linux environment, including suitable cloud-computing infrastructures such as Amazon EC2. A comprehensive manual, dependencies list, tutorial and sample data are also provided.

Given that data analysis has become an important bottleneck for the effectiveness of microbial ecology studies, the development of user-friendly computational tools remains a high priority. AXIOME represents an important step in this direction by automating multi-step bioinformatic analyses and enabling the customization of procedures to suit the diverse research needs of the microbial ecology community.

## Availability and requirements

**Project name:** AXIOME, Axiometic

**Project home page:**http://neufeld.github.com/axiome, http://neufeld.github.com/axiometic

**Operating system:** Linux (AXIOME), Platform independent (Axiometic)

**Programming language:** C, Vala, R, Python

**Other requirements:** QIIME (and dependencies therein), make, awk, mothur (optional), PANDAseq (optional); see documentation for a comprehensive list of optional dependencies, based on workflow requirements.

**License:** GPL v3

**Any restrictions to use by non-academics:** No

## Abbreviations

AXIOME: Automation, eXtension, and Integration Of Microbial Ecology; CPU: Central processing unit; GUI: Graphical user interface; MRPP: Multi-response permutation procedures; NMF: Non-negative matrix factorization; OTU: Operational taxonomic unit; PANDAseq: PAired-eND assembler for Illumina sequences; QIIME: Quantitative insights into microbial ecology; SSU: Small subunit

## Competing interests

The authors declare that they have no competing interests.

## Authors’ contributions

MDJL contributed to the design of AXIOME and manuscript preparation. APM designed and implemented AXIOME. MWH contributed to AXIOME and implemented Axiometic. AKB contributed to testing of AXIOME. JDN contributed to the design and coordination of the project, testing of AXIOME and manuscript preparation. All authors read and approved the final manuscript.
